# The effect of positive end-expiratory pressure on cardiac output and oxygen delivery during cardiopulmonary resuscitation

**DOI:** 10.1186/s40635-020-00330-2

**Published:** 2020-07-25

**Authors:** Yosef Levenbrown, Md Jobayer Hossain, James P. Keith, Katlyn Burr, Anne Hesek, Thomas Shaffer

**Affiliations:** 1 Division of Pediatric Critical Care, Nemours/Alfred I. duPont Hospital for Children, 1600 Rockland Road, Wilmington, DE 19803 USA; 2 Department of Pediatrics, Sidney Kimmel Medical School, Thomas Jefferson University, Philadelphia, PA USA; 3 Nemours Biomedical Research, Wilmington, DE USA; 4 Department of Applied Economics and Statistics, University of Delaware, Newark, DE USA; 5 Department of Respiratory Care, Nemours/Alfred I. duPont Hospital for Children, Wilmington, DE USA; 6 Nemours Biomedical Research/Research Lung Center, Wilmington, DE USA; 7 Department of Pediatrics, Lewis Katz School of Medicine at Temple University, Philadelphia, PA USA

**Keywords:** Cardiopulmonary resuscitation, PEEP, Cardiac output, Ventilation, Resuscitation

## Abstract

**Background:**

Positive end-expiratory pressure (PEEP) is used to optimize oxygenation by preventing alveolar collapse. However, PEEP can potentially decrease cardiac output through cardiopulmonary interactions. The effect of PEEP on cardiac output during cardiopulmonary resuscitation (CPR) is not known.

**Methods:**

This was a preclinical randomized, controlled, animal study conducted in an animal research facility on 25 Landrace-Yorkshire pigs. After inducing cardiac arrest, CPR was performed with LUCAS 3. During CPR, pigs were ventilated at a PEEP of 0, 5, 10, 15, 20 cmH_2_O (randomly determined via lottery) for 9 min. Cardiac output, obtained via ultrasound dilution, and PaO_2_ were measured, and oxygen delivery calculated for each PEEP.

**Results:**

A mixed-effects repeated-measures analysis of variance was used to compare the baseline value adjusted mean cardiac output, PaO_2_, and oxygen delivery between PEEP groups. Least significant difference test was used to conduct pairwise comparisons between PEEP groups. To determine optimum PEEP, Gaussian mixture model was applied to the adjusted means of cardiac output and oxygen delivery. Increasing PEEP to 10 and higher resulted in significant declines in cardiac output. A PEEP of 15 and higher resulted in significant declines in oxygen delivery. As PEEP was increased from 0 to 20, PaO_2_ increased significantly. Gaussian mixture model identified the 0–5 PEEP group as providing optimal cardiac output and oxygen delivery, with PEEP of 5 providing the highest oxygen delivery.

**Conclusions:**

A PEEP of 0–5 resulted in the optimal oxygen delivery and cardiac output during CPR, with PEEP of 5 resulting in higher oxygen delivery, and a slightly lower, statistically insignificant cardiac output than PEEP of 0.

## Background

Even though the importance of providing intermittent positive pressure ventilation during cardiopulmonary resuscitation (CPR) is recognized, the utilization of positive end-expiratory pressure (PEEP) in between breaths is a matter of debate.

Typically, when providing positive pressure ventilation, PEEP is used to prevent alveolar collapse, improve lung compliance, increase functional residual capacity, and maintain the adequate surface area of the lung for oxygen delivery (DO_2_) [1]. However, current CPR guidelines do not discuss whether PEEP should be used during CPR. A recent survey regarding how practitioners ventilate patients during CPR demonstrated a divergence of opinions as to whether or not PEEP is used, and how much PEEP is used during CPR [2]. Some believe, based on older studies, that during CPR, PEEP can potentially lead to a decline in cardiac output (CO) through a complex series of cardiopulmonary interactions [3, 4]. However, PEEP may be necessary to ensure adequate alveolar gas exchange during CPR. It has been shown that lack of PEEP use during CPR has been associated with hypercarbia and relative hypoxia compared with when PEEP is used [4, 5]. Some providers remain hesitant to supply any PEEP during CPR due to a concern that the potential decrease in CO caused by the use of PEEP will offset the benefit gained by improved gas exchange [2, 6]. Considering the contrast in PEEP effects on CO and PaO_2_, we hypothesized that there will be a clinically relevant PEEP level that optimizes CO and DO_2_ to the tissues during CPR. As such, we sought to evaluate the effect of PEEP on CO, PaO_2_, and DO_2_ during CPR to determine the optimum PEEP for CO, PaO_2_, and DO_2_ to the tissues during CPR.

## Methods

### Anesthesia and monitoring

This study was performed with 25 Landrace-Yorkshire pigs weighing 27–37 kg. The animals were purchased from the supplier on the day of the experiment and were not housed within our facility prior to the experiment. The experimental protocol was approved by the Nemours Animal Care and Usage Committee. The care and handling of the animals were in accord with the National Institutes of Health guidelines. The pigs received initial sedation with two intramuscular injections 10 min apart of 1 ml/kg of KAX, an anesthetic cocktail containing ketamine 23 mg/ml, acepromazine 0.58 mg/ml, and xylazine 0.8 mg/ml. Following the initial sedation, a left carotid intra-arterial catheter and right internal jugular central venous catheter were placed using standard cut-down techniques. The pigs were then intubated via midline tracheostomy using a cuffed 7.0–7.5 endotracheal tube. The animals were connected to a ventilator (Servo-I, Getinge, Wayne, NJ USA) and ventilated using volume control mode ventilation, with a tidal volume of 8 ml/kg, a rate of 20 breaths per minute, PEEP of 5 cmH_2_O, and FiO_2_ of 1.0. The ventilator breath rate was subsequently adjusted to maintain pH in the 7.35–7.45 range. The pigs then were given a bolus dose of propofol 1 mg/kg and ketamine 15 mg/kg and placed on infusions of propofol 3 mg/kg/h and ketamine 15 mg/kg/h. Bolus doses were repeated, and the continuous infusions were increased if the pig showed signs of pain, such as flinching, or a 10% increase in heart rate or blood pressure to a hoof pinch with a clamp. Intramuscular anesthesia (choice of medications and dosing) was based on a protocol used in our laboratory for prior studies. Intravenous anesthetic medications and dosing were based on published recommendations [7, 8]. The pigs were then given a 30-min stabilization period. Throughout the duration of the study, the pigs received continuous monitoring of heart rate, blood pressure, respiratory rate, and end-tidal carbon dioxide.

### Experimental protocol

The COstatus CO monitor (Transonic Systems, Ithaca, New York USA) was connected to the central venous line as well as the arterial line. The COstatus system measures CO using ultrasound dilution technology. This device has been validated, in humans as well as in a pig model, for CO measurements by comparison to thermodilution [9–13]. Baseline (pre-cardiac arrest) CO measurements and arterial blood gases were obtained (Nova Biomedical, Stat Profile Prime Analyzers, Waltham, MA USA). Bupivacaine 6–9 mg/kg was administered intravenously. This dose of bupivacaine has been shown to cause an irreversible cardiac arrest in a porcine model [14, 15]. Cardiac arrest was confirmed by looking for asystole on the three-lead cardiac monitor, as well as signs of no blood-flow (loss of pulsatile arterial line tracing, loss of pulse oximeter tracing, and loss of end-tidal CO_2_ tracing) for a full minute. After 1 min of cardiac arrest, a LUCAS 3 mechanical CPR compression device (Stryker, Kalamazoo, MI USA) was applied to the pig, and compressions were started at a rate of 100 compressions per minute.

Using a lottery system for randomization, the pig was then placed on one of five PEEP levels: 0, 5, 10, 15, and 20 cmH_2_O. The pig was maintained on each PEEP level for 9 min. Cardiac output was measured twice for each PEEP level, one at 5 min and a second measurement at 9 min, with the two values averaged together to give a CO for each PEEP level. Arterial blood gas was obtained at the end of the 9-min period for each PEEP level that the pig was on. The ventilator was then changed to the next randomly selected PEEP level. This process was repeated for all five PEEP levels. Oxygen delivery was calculated using the equation: DO_2_ = [(1.34 × hemoglobin × SpO_2_) + (0.0031 × PaO_2_)] × (CO × 10).

### Statistical analysis

Baseline physiologic characteristics including weight and cardiovascular parameters are summarized using mean and standard error of mean. A mixed-effects repeated-measures analysis of variance was used to compare the baseline value adjusted mean CO, PaO_2_, and DO_2_ between PEEP groups. Cardiac output, PaO_2_, and DO_2_ were used as the response variables; while piglet identification number was used as the random effect; and PEEP group, measurement time-points, and corresponding baseline values were used as fixed effects in each model. The least significant difference test was used to conduct pairwise comparisons between the PEEP groups. Model assumptions were checked before analysis. To decide the optimum PEEP level, we applied the Gaussian mixture model on the adjusted means of CO and DO_2_. All tests were two-tailed at an overall level of significance of 0.05. Statistical software SAS version 9.4 (Cary, NC USA) was used for the analysis. A sample size of 25 piglets was used in this study based on an a priori power (80%) estimation to detect the significant difference (overall) in post-CPR mean CO and DO_2_ between PEEP levels. Post-CPR outcome variables were measured at five time points for each piglet. The PEEP levels were assigned to time points randomly.

## Results

Twenty-five piglets with mean (standard error or mean) weight of 34.8 (0.41) kg were used in the study. Baseline cardiovascular parameters including CO, PaO_2_, and DO_2_ are summarized in Table 1.

**Table 1:** Baseline physiologic characteristics

Variables	Mean (SEM)
Blood chemistry	
pH	7.39 (0.01)
PCO_2_ (mmHg)	40.94 (0.79)
PaO_2_ (mmHg)	397.09 (13.14)
Hemoglobin (gm/dL)	8.39 (0.24)
Oxygen saturation (%)	100 (0.04)
Oxygen delivery (ml/min)	407.7 (31.0)
Cardiovascular parameters	
BP systolic (mmHg)	85.12 (2.77)
BP diastolic (mmHg)	58.08 (2.29)
Heart rate (beats/min)	95.52 (4.32)
Cardiac output (l/min)	3.21 (0.22)

*BP* blood pressure, *SEM* standard error of mean

**Fig. 1:**
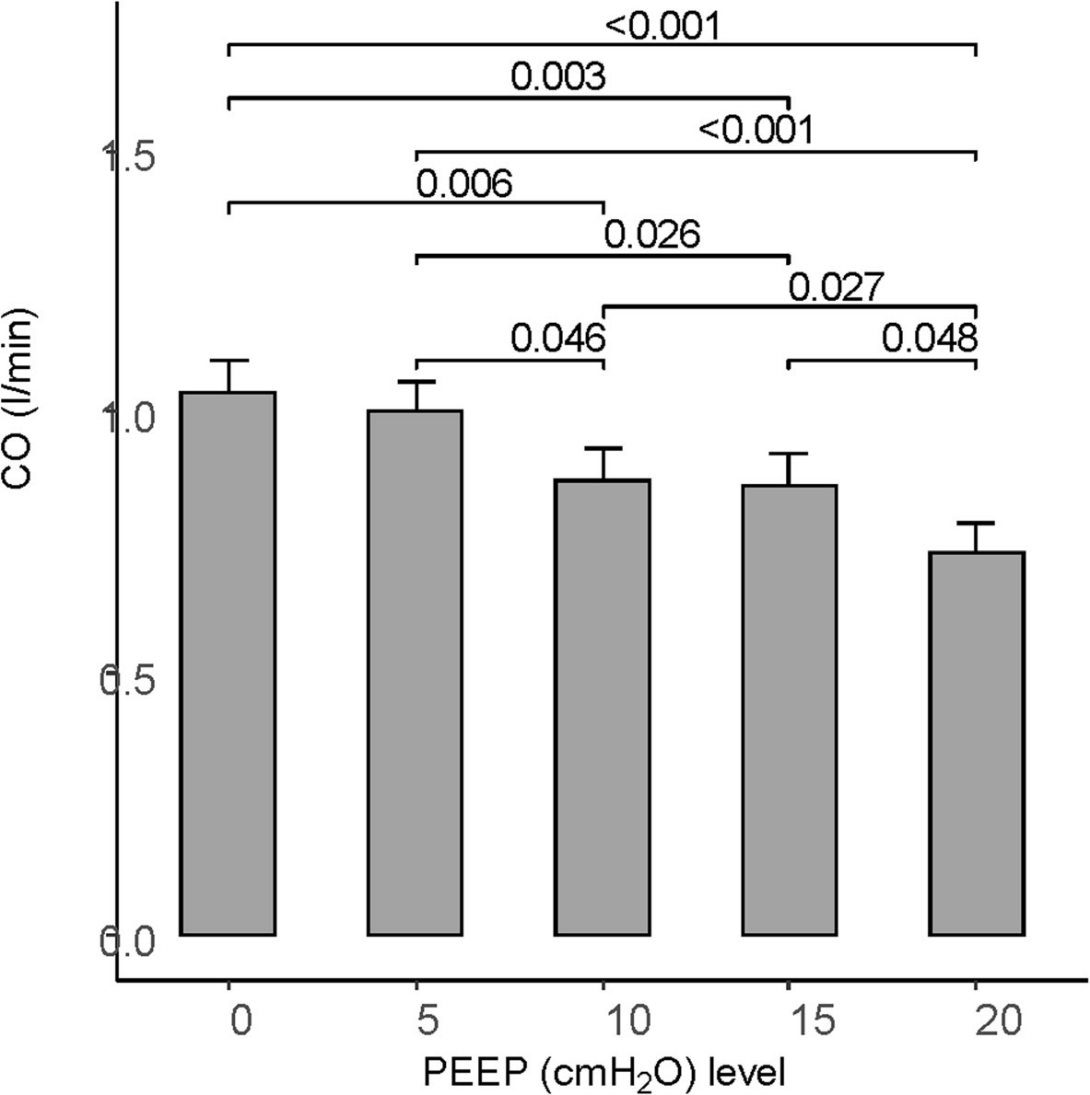
Comparison of cardiac output for the different positive end-expiratory pressure (PEEP) levels during cardiopulmonary resuscitation. CO cardiac output

The estimated means of CO for each of the five PEEP levels during CPR, after adjustment for baseline CO and time of the measurements, are summarized in Table 2 and shown in Fig. 1. The *P* value <0.001 indicates a significant overall mean difference of CO among PEEP levels, such that as the PEEP increases, CO decreases monotonically. Pairwise comparisons of CO for the different PEEP levels are listed in Table 3. As demonstrated, there is no significant drop in CO as PEEP is increased from 0 to 5. However, there is a significant drop in CO as PEEP is increased from 0 to 10 cmH_2_O (*P* = 0.006) and from 5 to 10 cmH_2_O (*P* = 0.046). As noted previously, CO continues to decrease monotonically as PEEP is further increased. No significant difference in mean CO was observed between PEEP levels 10 and 15 cmH_2_O. Finally, it is most noteworthy that mean CO at PEEP = 20 cmH_2_O is substantially smaller than that at any other level. Note that at a PEEP of 5 cmH_2_O during CPR, CO was approximately 30% of baseline CO (also at a PEEP of 5 cmH_2_O).

**Table 2:** Primary outcomes: mean CO, PaO_2_, and oxygen delivery after adjustment for time and baseline values

PEEP level	Cardiac output (l/min)	PaO_**2**_ (mmHg)	Oxygen delivery (ml/min)
0	1.04 (0.06)	154.54 (24.75)	107.72 (6.77)
5	1 (0.06)	201.83 (24.79)	110.78 (6.79)
10	0.87 (0.06)	205.53 (24.72)	100.9 (6.77)
15	0.86 (0.06)	221.81 (24.74)	94.6 (6.77)
20	0.73 (0.06)	266.73 (24.76)	81.95 (6.79)
*P* value	<.001	<.001	<.001

Note: *P* value indicates the significance to compare the overall mean differences across PEEP levels. *CO* cardiac output, *PEEP* positive end-expiratory pressure

**Table 3:** Pairwise comparisons of main outcomes across positive end-expiratory pressure levels

PEEP level pairs		Cardiac output (l/min)		PaO_**2**_ (mmHg)		Oxygen delivery (ml/min)	
		MeanDiff (SEM)	***P***	MeanDiff (SEM)	***P***	MeanDiff (SEM)	***P***
0	5	0.05 (0.06)	.45	−47.29 (14.48)	.002	−3.05 (6.69)	.649
0	10	0.17 (0.06)	.006	−51 (13.99)	<.001	6.82 (6.46)	.294
0	15	0.18 (0.06)	.003	−67.27 (13.91)	<.001	13.12 (6.43)	.044
0	20	0.31 (0.06)	<.001	−112.19 (13.98)	<.001	25.77 (6.46)	<.001
5	10	0.12 (0.06)	.046	−3.7 (14.14)	.794	9.88 (6.54)	.135
5	15	0.14 (0.06)	.026	−19.98 (13.92)	.155	16.18 (6.44)	.014
5	20	0.26 (0.06)	<.001	−64.9 (13.61)	<.001	28.82 (6.31)	<.001
10	15	0.01 (0.06)	.843	−16.28 (14.21)	.255	6.3 (6.54)	.338
10	20	0.14 (0.06)	.027	−61.2 (14.58)	<.001	18.95 (6.71)	.006
15	20	0.13 (0.06)	.048	−44.92 (14.97)	.003	12.65 (6.86)	.068

*MeanDiff* mean difference, *PEEP* positive end-expiratory pressure, *SEM* standard error of mean

**Fig. 2:**
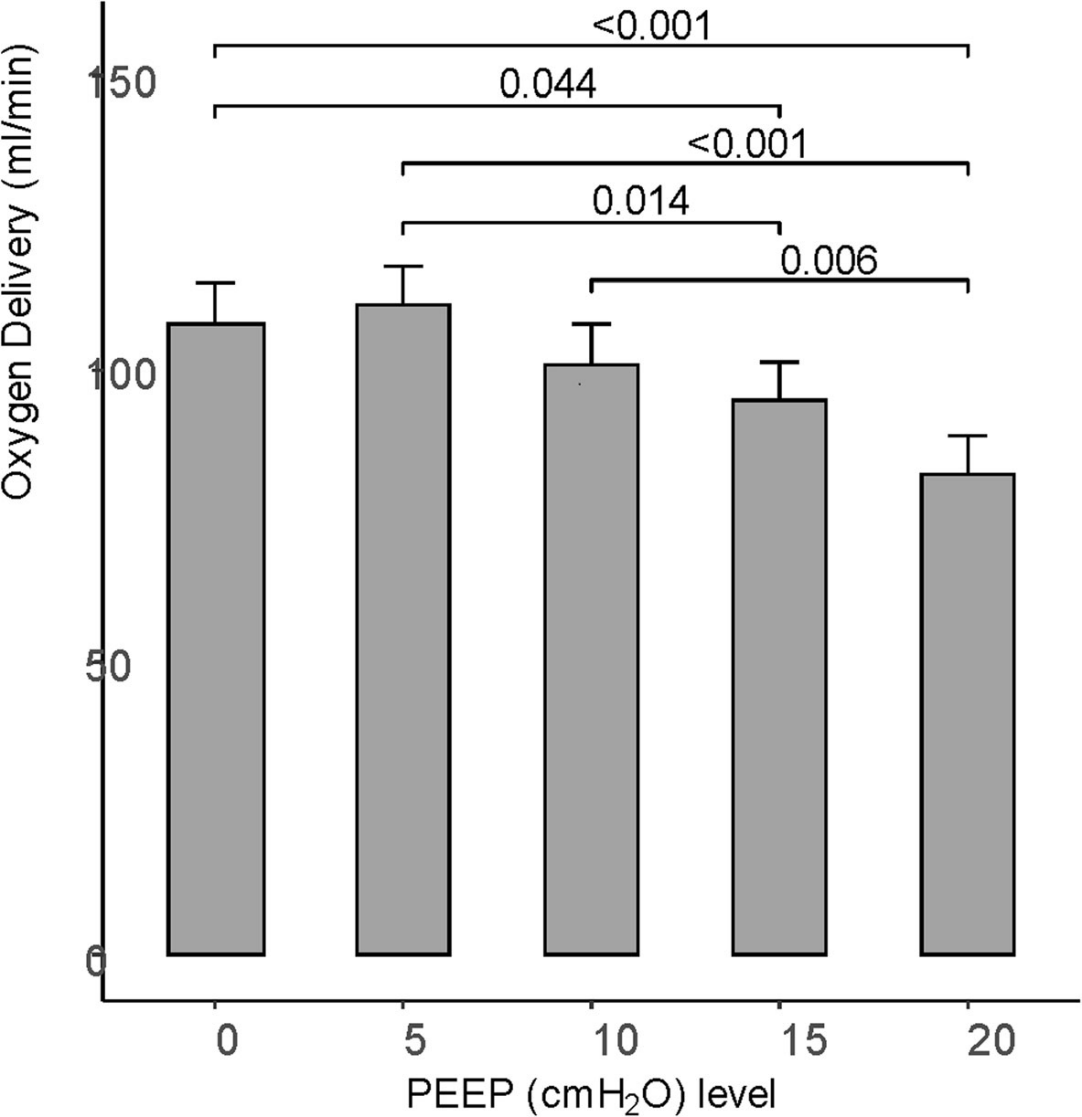
Comparison of oxygen delivery for the different positive end-expiratory pressure (PEEP) levels during cardiopulmonary resuscitation

The estimated mean DO_2_ for all PEEP levels, after adjustment for baseline values as well as the time the measurements were obtained, are summarized in Table 2 and shown in Fig. 2. The *P* value of <0.001 indicates a significant overall difference in DO_2_, such that as PEEP increases, DO_2_ significantly decreases with the exception of 0 to 5 cmH_2_O PEEP, which resulted in a slight nonsignificant increase in DO_2_. As shown in Fig. 2 and summarized in Table 3, pairwise comparisons of DO_2_ for the different PEEP levels further demonstrate that compared with PEEP of 0 and 5 cmH_2_O, a PEEP of 15 and 20 cmH_2_O resulted in a significant decline in DO_2_. A PEEP of 10 did not result in a significant decline in DO_2_ when compared with either a PEEP of 0 or 5 cmH_2_O. As shown, mean DO_2_ was maximum for PEEP of 5 cmH_2_O.

**Fig. 3:**
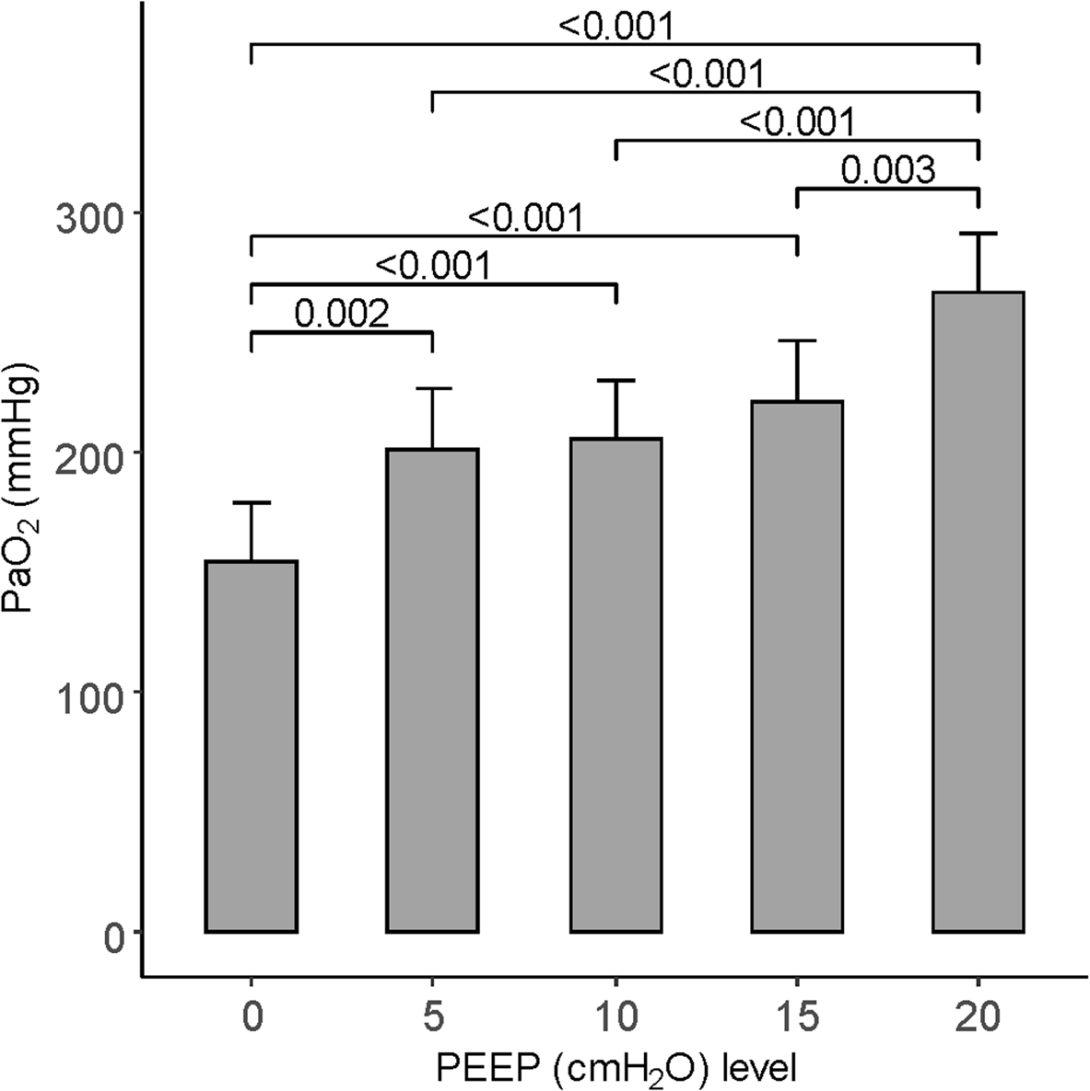
Comparison of PaO_2_ for the different positive end-expiratory pressure (PEEP) levels during cardiopulmonary resuscitation

The estimated mean PaO_2_ after adjustment for baseline PaO_2_ and time is summarized in Table 2 and illustrated in Fig. 3. After adjustment for the time of measurements and the baseline PaO_2_, there is a significant difference in mean PaO_2_ between PEEP levels demonstrating that as PEEP increases, PaO_2_ monotonically increases (*P* value <0.001). However, as demonstrated in Table 3, when a pairwise comparison of PaO_2_ for the different PEEP levels is performed, increases in PEEP above 0 result in significant increases in PaO_2_: PEEP 0–5 cmH_2_O (*P* = 0.002), PEEP 0–10 cmH_2_O (*P* < 0.006), PEEP 0–15 cmH_2_O (*P* < 0.003), and PEEP 0–20 cmH_2_O (*P* < 0.001). Compared with PEEP of 5 cmH_2_O, PEEP of 15 and 20 cmH_2_O have a significant increase in PaO_2_.

Application of the Gaussian mixture model on adjusted means of CO and DO_2_ (to determine the optimal PEEP level) demonstrate three distinct groups of PEEP levels with 0 and 5 cmH_2_O in one group, 10 and 15 cmH_2_O in another group, and PEEP level 20 cmH_2_O as a distinct group, with PEEP 0–5 cmH_2_O as the optimal PEEP level for CO and DO_2_.

## Discussion

When lung compliance is poor such as in patients with pneumonia or bronchiolitis or when there is excess lung fluid for example with pulmonary edema, the critical closing pressure is increased, and lungs become more likely to collapse. During mechanical ventilation, by maintaining the PEEP above the critical closing pressure, alveolar collapse can be prevented [1].

Through a complex series of cardiopulmonary interactions, PEEP can potentially have a direct impact on CO. The blood in the venous system relies on the pressure difference generated by the venous blood, the mean systemic pressure, and the right atrium to promote the forward flow of blood back to the heart. This difference between the mean systemic pressure and the right atrial pressure is the driving force for the preload [16–18]. When PEEP is used, the increase in alveolar pressure is transmitted to the entire thorax, potentially increasing the right atrial pressure and thereby reducing the pressure difference between the mean systemic pressure and the right atrial pressure. If the decrease in this pressure gradient is significant enough, it can result in decreased venous return to the heart, decreasing cardiac output [19, 20]. The exact impact that this effect has is questionable, as a study by Jellinek et al. demonstrated that positive pressure increases both the right atrial pressure as well as the mean systemic pressure proportionally, resulting in no change in the mean systolic pressure-right atrium pressure gradient [21]. The use of too much PEEP can over-distend alveoli resulting in mechanical compression of the pulmonary capillaries, increasing the right ventricular (RV) afterload. An increase in the RV afterload can over-distend the RV, causing bowing of the ventricular septum into the left ventricular (LV), thereby further decreasing the volume of the LV, decreasing LV filling, and reducing CO. On the left side of the heart, PEEP can shift the LV pressure-volume curve to the left, indicating a decrease in LV distensibility. Thus, given that during CPR blood flow and venous return are already compromised as optimal CPR generates only 15% to 25% of normal CO [22], these effects of PEEP on CO can potentially diminish an already severely compromised CO.

Conversely, although an inappropriate amount of PEEP can have a detrimental effect on CO, using an appropriate amount of PEEP can potentially augment CO. Increasing the intrathoracic pressure can decrease the LV afterload, thereby improving the CO, especially in the setting of a poorly functioning LV. Additionally, the correct amount of PEEP can optimize peripheral vascular resistance, thereby improving LV preload [1, 19, 20].

The use of PEEP can potentially play a significant role in the ability to ventilate patients receiving CPR. Studies performed by the Cardiac Arrest and Ventilation International Association for Research Group demonstrated that in a cadaver model and a bench model of CPR, as well as in a clinical study analyzing capnograms of intubated patients receiving CPR, intrathoracic airway closure occurs in patients receiving CPR, which can limit ventilation. This airway closure was mitigated by the use of PEEP up to 10 cmH_2_O, which also caused some degree of ventilation to occur with the oscillations of air generated by the change in intrathoracic pressure that occurs during the compression and decompression phase of chest compressions [23, 24]. The effect that using no PEEP potentially has on oxygenation and DO_2_ to the tissues during CPR is also unknown.

There are currently no studies directly evaluating the effect of PEEP on CO during CPR. There is also currently no consensus whether or not PEEP should be applied during CPR, and if used, how much PEEP should be applied. The aim of this study was to evaluate the effect of PEEP on CO and DO_2_ during CPR and to determine the ideal PEEP to maximize DO_2_ by augmenting both CO and arterial oxygen concentration during CPR.

The results of this study demonstrate that as PEEP is increased from 0 to 20 cmH_2_O, there is a significant decline in CO and DO_2_. Increasing the PEEP from 0 to 5 cmH_2_O results in a slight, statistically insignificant, decrease in CO, and increase in DO_2_. Further increases in PEEP to 10 cmH_2_O and above result in significant drops in CO. Even compared with PEEP of 5 cmH_2_O, PEEP of 10 cmH_2_O showed a significant decline in CO. For DO_2_, compared with both PEEP of 0 and 5 cmH_2_O, once PEEP 15 cmH_2_O and higher is reached, there is a statistically significant drop in DO_2_. In evaluating the effect of PEEP on PaO_2_ during CPR, as PEEP is increased from 0 to 20 cmH_2_O, there is a significant increase seen in PaO_2_. Compared with a PEEP of 0 cmH_2_O, PEEP of 5, 10, and 15 cmH_2_O all had significantly higher PaO_2_. Compared with PEEP of 5 cmH_2_O, only PEEP of 20 cmH_2_O had a significantly higher PaO_2_.

Using the Gaussian mixture model on adjusted means of CO and DO_2_, there were three groups of homogeneous PEEP that were identified: 0–5, 10–15, and 20 cmH_2_O. PaO_2_ was not included in this analysis because even the lowest PEEP had a PaO_2_ of 154, which is physiologic, representing an oxygen saturation of 100% and is likely adequate for CPR. In addition, the importance of PaO_2_ is likely to be in the amount of oxygen delivered to the tissues during CPR, making DO_2_ the more important variable. Based on these results, assuming that the lungs are not acutely ill or poorly compliant, our results demonstrate that the 0–5 cmH_2_O PEEP group provides optimal CO and DO_2_, with PEEP of 5 cmH_2_O providing the highest DO_2_ overall, with an insignificant difference in CO between PEEP of 0 cmH_2_O and 5 cmH_2_O. Thus, based on these results, it appears that PEEP of 5 cmH_2_O would be the optimal PEEP for ventilating patients during CPR.

This study has a number of limitations. Most CPR studies are done on animals or other non-human models, and the porcine model is commonly used as a model for cardiac arrest because the physiology approximates that of humans [14]. However, it has to be acknowledged that there are differences in cardiovascular physiology between humans and pigs, such as different thorax geometry [24], which makes it an imperfect model for CPR physiology in humans. Also, the outcomes in this study are meant to evaluate cardiovascular parameters during CPR, with the goal of optimizing organ perfusion during CPR. However, there is no evidence that following the conclusions in this study will directly lead to a better outcome in cardiac arrest patients. However, we do feel that to give providers the best chance at successfully resuscitating a patient, CPR must be optimized, and adjusting the PEEP to achieve optimal oxygen delivery is a potential area of optimization. Ideally, these findings should be verified with a proper trial in humans; however, such a trial may be extremely difficult to design and implement. Although CPR is not typically performed with patients on a ventilator, PEEP adjustments can be made through the PEEP valve on a bag-valve-mask device. Finally, the goal of this study was to isolate the effect of PEEP on CO. To accomplish this, slight adjustments were made to optimize physiologic parameters, such as allowing the animal to tolerate 60 min of CPR, which is not typically done when performing CPR on humans. For example, the respiratory rate was higher than the 10 breaths per minute currently recommended during continuous compression CPR to achieve and maintain a physiologic pH prior to and during CPR. Along the same lines, the decision was made to maintain the animal on each PEEP level for 9 min, with 1 min in between to draw the arterial blood gas and reconnect cardiac output monitor to maintain the entire duration of CPR less than 1 h. We felt that if the compressions were to continue for more than an hour, the animals would be less stable for the final PEEP levels, which would introduce another variable into the equation. By keeping the total compression time less than 1 h, we felt that the animal would be able to tolerate the entire course of compressions, whereas extending the overall time of compressions would have risked greater instability towards the end, compared with the beginning. Using a two-way analysis of variance paradigm for the statistical analysis took into account PEEP level as well as time duration in the final analysis. If the animals were unstable for the final PEEP levels, this would have added an additional variable that would have been difficult to account for. In addition, after initiating cardiac arrest and during the 60 s prior to initiation of chest compression, the pigs were ventilated using a PEEP of 5 cmH_2_O to avoid alveolar collapse prior to the implementation of the study protocol, despite this differing from what occurs in typical cardiac arrest patients, who are not receiving PEEP when they go into cardiac arrest. We feel that these do not detract from the results of this study, as the goal of the study is to look at the effect PEEP has on CO, which this study certainly does.

## Conclusions

In this study, analyzing the effect of PEEP on CO, PaO_2,_ and DO_2_ during CPR, PEEP of 0–5 cmH_2_O resulted in the optimal DO_2_ and CO, with PEEP of 5 resulting in a higher DO_2_, and a slightly lower, statistically insignificant, CO than PEEP of 0. Thus, based on the contrasting effects of PEEP on CO, PaO_2_, and DO_2_, the results of our study would suggest a PEEP of 5 cmH_2_O is the optimum PEEP level for performing CPR, at least in this model of CPR for cardiac arrest.

## Data Availability

Datasets used and/or analyzed during the current study are available from the corresponding author on reasonable request.

## Abbreviations

CO: Cardiac output
CPR: Cardiopulmonary resuscitation
DO_2_: Oxygen delivery
LV: Left ventricular
PEEP: Positive end-expiratory pressure
RV: Right ventricular
